# Effect of an accelerating metro cabin on the diffusion of cough droplets

**DOI:** 10.1038/s41598-024-64026-3

**Published:** 2024-06-19

**Authors:** Ge Peng, Fang Liu

**Affiliations:** 1 Institute of Geomatics and Urban Spatial Informatics, Beijing University of Civil Engineering and Architecture, Beijing, 100044 China; 2 Key Laboratory of Urban Spatial Information, Ministry of Natural Resources, KLUSI, Beijing, 100044 China

**Keywords:** coronavirus disease, Metro, Accelerated cabin, Internal**-**induced airflow, Inertial force, Environmental sciences, Diseases

## Abstract

Coronaviruses being capable of spreading through droplet contamination have raised significant concerns regarding high-capacity public rail transport, such as the metro. Within a rapidly moving railcar cabin, the internal airflow lags behind the bulkhead, generating internally induced airflow that accelerates droplet dispersion within a non-inertial reference system. This study investigates the impact of acceleration on the diffusion of cough droplets of varying sizes using computational fluid dynamics. The modified k–ε equation in ANSYS® Fluent was utilized to simulate droplet diffusion under different body orientations by adjusting the inertial force correction source term. Results indicate that droplets in the middle size range (50–175 μm) are primarily influenced by inertial forces, whereas smaller droplets (3.5–20 μm) are predominantly controlled by air drag forces. Regardless of facial orientation, the outlet of high-capacity public rail transport poses the highest risk of infection.

## Introduction

Complex urban transportation systems, comprising airplanes, metros, and buses, facilitate extensive travel convenience but also pose a significant risk of cross-infection among travelers within intricate infection networks^1–3^. Research indicates that mediums such as metros, buses, and ships are pivotal in the outbreak and spread of epidemics^4–6^. The urban metro system operates on a fixed network and timetable, experiencing phases of acceleration, constant velocity, and deceleration during the journey between two adjacent stations. At network nodes, newly infected travelers may be introduced into other branches of the network. Based on these observations, the metro cabin, influenced by internally generated airflow during acceleration and deceleration, was selected as the focus for studying the dispersion of cough droplets of varying sizes^7^.

**Figure 1:**
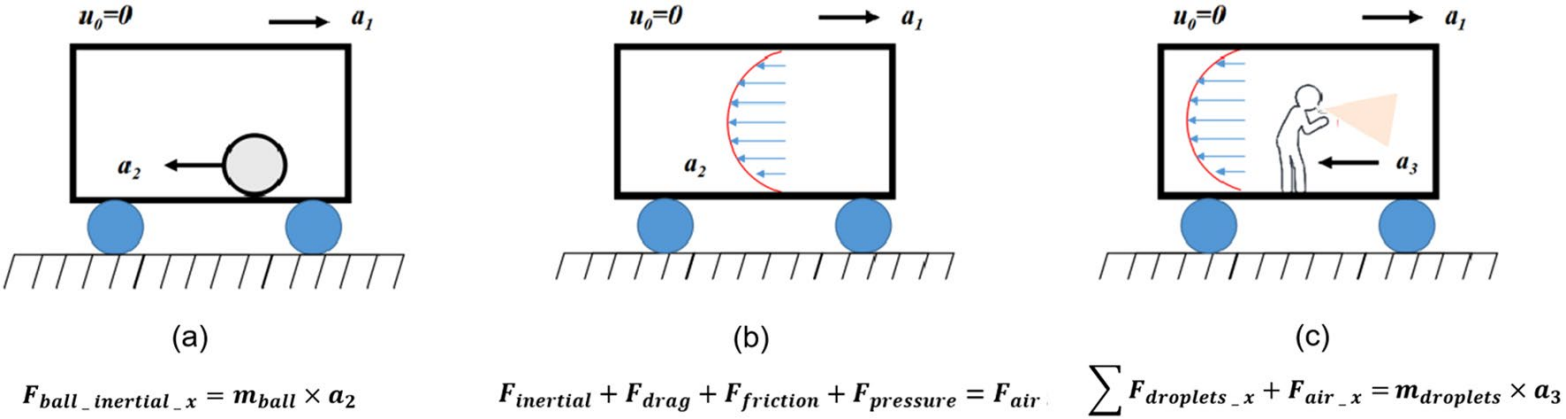
Multiple effects of inertial and drag forces on droplet emission (**a**) Rigid ball motion in the accelerated motion cabin (**b**) Internal induced flow motion in accelerated motion cabin (**c**) Inertial effects of cough droplet jets.

The roles of inertial, drag, and pressure forces on droplet emission are critical yet understudied factors. This study investigates how these forces affect the diffusion of cough droplets across different sizes within a moving metro cabin, including the simulation and practical application of safe distances for standing passengers (Fig. 1). The subway train’s dynamic movements—linear accelerations, abrupt stops, and the wall-gas drag effect—create airflow that facilitates droplet spread. The inertia of cough droplets alters their trajectories, affecting infection rates. Variations in air pressure during these phases enhance droplet dispersion, significantly increasing infection risks^8^. Consequently, evaluating the efficacy of social distancing to control virus spread in a semi-sealed, non-inertial metro setting becomes essential.

Primary emission sources of pathogen-laden droplets, such as coughing, sneezing, and talking, generate high-velocity, warm, humid microenvironments that can propel droplets up to 25 m in just 0.5 s^9,10^. Droplet dispersion is influenced by size, velocity, airflow, and surface characteristics^11^. Droplets of various sizes carry pathogens over different distances, depending on their initial momentum and size. For example, sneezing can generate airflow velocities of 25–40 m/s, producing up to 10,000 droplets, whereas coughing may produce jet velocities of 6–22 m/s, resulting in up to 3,000 droplets of varying sizes laden with pathogens^12^. Larger droplets (> 50 μm) quickly settle due to gravity, while smaller droplets (≤ 20 μm) can remain airborne for extended periods^11,13,14^, eventually forming droplet nuclei composed of proteins and pathogens. These nuclei, floating as aerosols, elevate the risk of airborne transmission and potential infection^10,15–17^.

While masks are commonly utilized to prevent droplet transmission, they do not provide complete protection. In response to ongoing epidemics, countries have adopted measures such as mask mandates, vaccinations, and maintaining safe distances; however, these precautions do not assure absolute virus protection. Even after vaccination, risks persist due to the emergence of "immunity escape variants." Establishing a universally applicable safe distance in dynamic environments is challenging, with differing standards across regions—for instance, Australia and certain Asian countries implement varying safe distance criteria. Therefore, understanding airflow's impact on droplet spread and establishing guidelines for safe, healthy semi-enclosed moving transportation environments are crucial.

Despite extensive research on droplet dynamics in various settings, there remains a significant gap concerning the impact of interior-induced airflow on cough droplet diffusion within moving transportation environments, such as metro cabins. This study seeks to bridge this gap by leveraging computational fluid dynamics (CFD) simulations to explore how acceleration-induced airflow affects droplet dispersion.

Previous research has successfully utilized CFD simulations to model droplet dispersion, thermal exchanges, and mass transfers during respiratory events like coughing and sneezing in static settings^18–20^. Recent advancements have extended these simulations to dynamic scenarios, including the study of cough droplet trajectories in a lecture hall under various ventilation strategies during the COVID-19 pandemic^21^.

Research also extends into the impact of droplets in diverse dynamic environments, such as indoor and outdoor settings and different modes of transportation. Blocken et al. (2020) analyzed droplet transmission between runners in outdoor settings^22^, while Hetherington et al. (2021) examined how droplets from motorcyclists affect passengers^23^. Li et al. (2021) explored droplet dispersion on escalators at varying speeds and angles^24^. Furthermore, Guan et al. (2014) utilized dynamic meshing and Lagrangian particle tracking methods to assess cough particle dynamics in indoor environments with moving subjects^25^. There are also studies of droplet dispersion in various types of transportation, such as buses, coaches, airplanes, high-speed railways, etc^5,6,26,27^.Despite these advances, studies specifically focusing on cough droplet dispersion under the dual influence of inertial and drag forces in non-inertial moving systems remain scarce. Talaee et al. (2019) measured velocity distributions in metro trains to estimate airflow dynamics, yet comprehensive pathogen spread studies are lacking^28^.

Therefore, this study aims to investigate the effect of induced airflow doubled with the inertial force on the diffusion of human cough droplets due to the accelerated motion of the metro cabin. Specifically, this study has three objectives. First, the modified k-ε equation will be proposed in computational fluid dynamics by modifying the User Defined Functions (UDF) for ANSYS Fluent^®^ inertial force correction source term. Second, the trajectory of cough droplets affected by the integrated impact of inertial force, the air drag force and the pressure force considering the linear acceleration of the cabin will be validated. Third, three comparative experiments will be organized, including scenarios with and without the injection of airflow in the cabin during the one-station period (acceleration-constant velocity-deceleration phases) and scenarios in three different orientations (facing the front (inlet), facing the rear (outlet), and facing the side), see Fig. 5. The results of this study will be valuable for calculating the infection rate and detecting safe positions in a non-inertial reference system. Specifically, the analysis of the diffusion trajectory and escape time of cough droplets will provide insights into the potential transmission of respiratory diseases in semi-enclosed moving transportation environments. By investigating the combined effects of induced airflow and inertial force on droplet dispersion, this study will advance the understanding of the complex dynamics of droplet diffusion in dynamic transportation environments and provide guidelines for creating a safe and healthy transportation environment for commuters.

## Material and methods

The Computational Fluid Dynamics (CFD) method was utilized to simulate induced airflow and droplet dispersion within a subway train cabin using ANSYS Fluent commercial software (Version 21.0). To accurately represent the metro cabin and the airflow patterns within it, the Reynolds-Averaged Navier–Stokes (RANS) algorithm was employed to solve the airflow model^29^. Additionally, the trajectories of droplets of various sizes were tracked using the Euler–Lagrange method, enabling a detailed analysis of their dispersion dynamics.

## Governing equations

The equations governing airflow and contaminant dispersion in the current cabin model solved by the ANSYS FLUENT V21.0 software are as follows:

The mass conservation (continuity) equation:

1 $$\begin{array}{*{20}c} {\frac{\partial \rho }{{\partial t}} + \nabla .\left( {\rho \vec{V}} \right) = 0} \\ \end{array}$$

where $\rho$ is the in-cabin air density, $t$ is the time, and $\vec{V}$ is the flow velocity vector.

- The momentum conservation equation:
  2 $$\begin{array}{*{20}c} {\frac{\partial }{\partial t}\left( {\rho \vec{V}} \right) + \nabla .\left( {\rho \vec{V}\vec{V}} \right) = - \nabla p + \nabla .\left( {\overline{\overline{\tau }}} \right) + \rho \vec{g} + \rho \vec{a}_{cabin} } \\ \end{array}$$
  where $p$ is static pressure, $\overline{\overline{\tau }}$ is the stress tensor, and $\overrightarrow{g}$ and ${\overrightarrow{a}}_{cabin}$ are the gravitational and cabin accelerations, respectively.
- The energy equation:
  3 $$\begin{array}{*{20}c} {\frac{\partial }{\partial t}\left( {\rho E} \right) + \nabla .\left( {\vec{V}\left( {\rho E + p} \right)} \right) = \nabla \cdot \left( {k_{eff} \nabla T - \mathop \sum \limits_{j} h_{j} \vec{J}_{j} + \left( {\overline{\overline{\tau }}_{eff} \cdot \vec{V}} \right)} \right) + S_{h} \# } \\ \end{array}$$
  where ${k}_{eff}$ is the effective conductivity=$k+{k}_{t}$ (${k}_{t}$ is the turbulent thermal conductivity), $T$ is the temperature, ${\overrightarrow{J}}_{j}$ is the diffusion flux of species $j$, and ${S}_{h}$ is an additional volumetric heat source (e.g., passenger bodies). $E$ represents the total energy per unit mass of the gas.
- Turbulence kinetic energy ($k$) and turbulence kinetic energy dissipation rate ($\varepsilon$) equations (renormalization group [RNG] $k-\varepsilon$ model):
  4 $$\begin{array}{c}\frac{\partial }{\partial t}\left(\rho k\right)+\frac{\partial }{\partial {x}_{i}}\left(\rho k{u}_{i}\right)=\frac{\partial }{\partial {x}_{j}}\left({\alpha }_{k}{\mu }_{eff}\frac{\partial k}{\partial {x}_{j}}\right)+{G}_{k}+{G}_{b}-\rho \varepsilon +{S}_{k}\#\end{array}$$
  5 $$\begin{array}{c}\frac{\partial }{\partial t}\left(\rho \varepsilon \right)+\frac{\partial }{\partial {x}_{i}}\left(\rho \varepsilon {u}_{i}\right)=\frac{\partial }{\partial {x}_{j}}\left({\alpha }_{\varepsilon }{\mu }_{eff}\frac{\partial \varepsilon }{\partial {x}_{j}}\right)+{C}_{1\varepsilon }\frac{\varepsilon }{k}\left({G}_{k}+{C}_{3\varepsilon }{G}_{b}\right)-{C}_{2\varepsilon }\rho \frac{{\varepsilon }^{2}}{k}-{R}_{\varepsilon }+{S}_{\varepsilon }\#\end{array}$$
  where ${\alpha }_{k}$ and ${\alpha }_{\varepsilon }$ are the inverse effective Prandtl numbers for $k$ and $\varepsilon$, respectively; ${S}_{k}$ and ${S}_{\varepsilon }$ are the user-defined source (or sink) terms; and ${C}_{1\varepsilon }$, ${C}_{2\varepsilon }$, and ${C}_{3\varepsilon }$ are constants defined by the RNG $k-\varepsilon$ model theory. In addition, ${G}_{k}$ represents the generation (or consumption) of turbulence kinetic energy due to the mean velocity gradients, and ${G}_{b}$ is the generation (or consumption) of turbulence kinetic energy due to buoyancy, which is formulated using the standard gradient diffusion hypothesis as,
  6 $$\begin{array}{c}{G}_{b}=-{g}_{i}\frac{{\mu }_{t}}{\rho P{r}_{t}}\frac{\partial \rho }{\partial {x}_{i}}\#\end{array}$$
  where ${g}_{i}$ is the component of the gravitational vector in the $i$ th direction, ${\mu }_{t}$ is the turbulent viscosity, and $P{r}_{t}$ is the turbulent Prandtl number.

### Geometry

#### Geometric model

**Figure 2:**
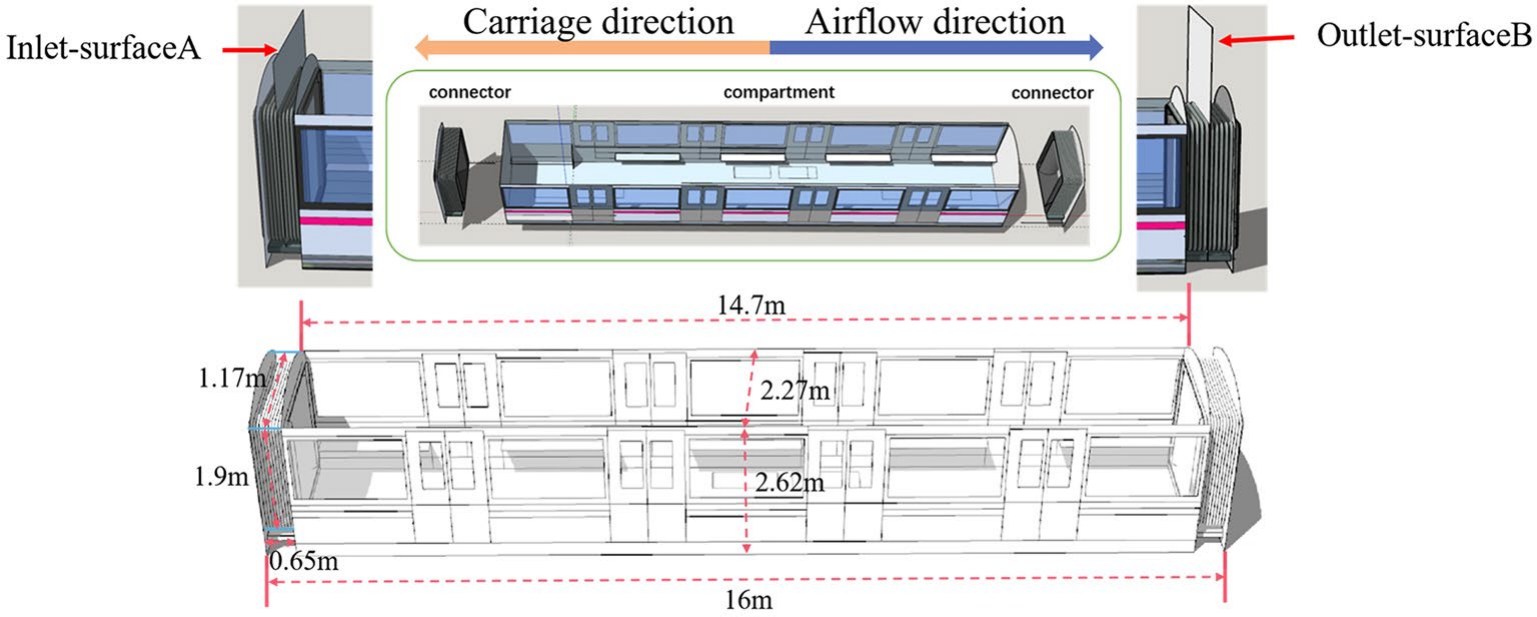
Cabin structure display and geometry.

The geometric model employed in this study was constructed based on the dimensions of a typical metro cabin, as specified by Talaee et al. (2019)^28^. The total length of a single cabin was set at 16 m, with specific dimensions and cabin composition detailed in Fig. 2 and Table 1. The airflow inlets and outlets were positioned at the midpoint of the carriage joint between two adjacent sections. The cabin's movement was depicted from right to left, indicated by the orange arrows in Fig. 2. Due to the constant alternation between acceleration, constant velocity, and deceleration phases, the airflow inside the cabin was pressurized, creating an induced flow whose direction varied over time. This directional change of the induced airflow is documented in Table 2, where 'a' represents acceleration, and 's' indicates the maximum constant velocity.

**Table 1:** Geometry of carriage.

Model	Compartment (L * W * H)	Cabin (L * W * H)
Size (m)	14.7 * 2.27 * 2.62	16 * 2.27 * 2.62

**Table 2:** Internal induced airflow direction.

Cabin phase	Airflow phase	Airflow direction
Acceleration phase (0–25s) a = + 0.9 m/s^2^	Acceleration phase (0–35s)	Opposite the direction of the cabin movement
Constant speed phase (25–45s) a = 0	Constant speed phase (35–45s)	Keep the same speed as the cabin (21 m/s)
Deceleration phase (45–75s) a = − 0.9 m/s^2^	Deceleration phase (45–75s)	Same direction as the cabin movement

**Figure 3:**
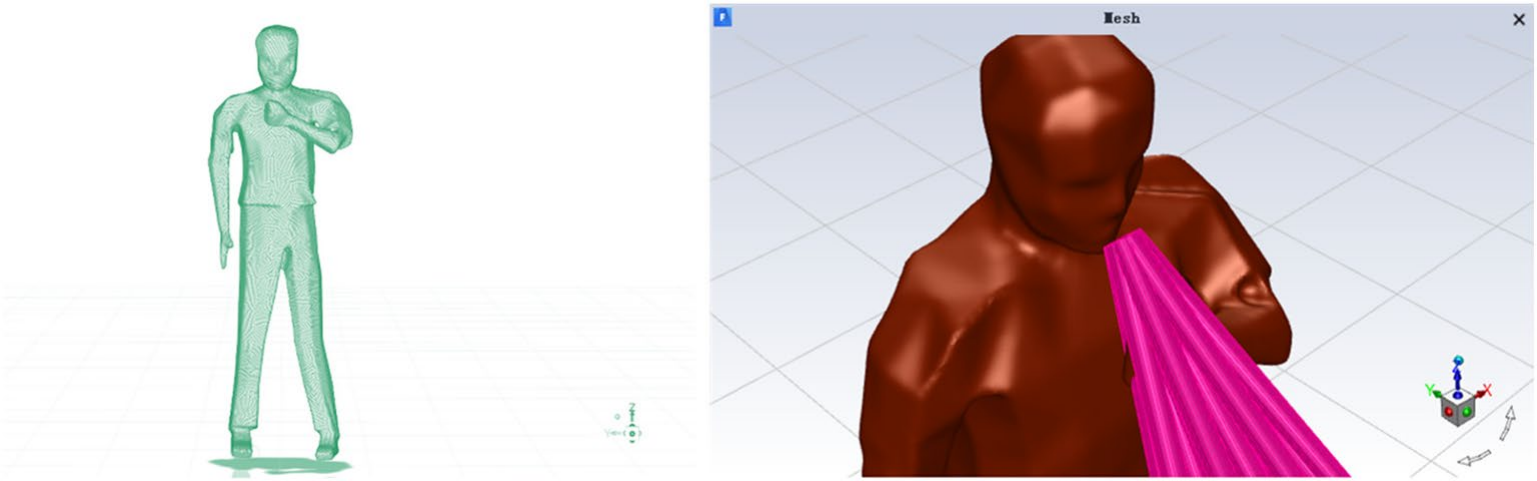
Mannequin geometry.

To model the dispersion of droplets, it was assumed that a single infected person was standing at the center of the compartment, shielding their mouth with their left hand, as illustrated in Fig. 3. Droplets were considered to be exhaled from the mouth of the infected person, covering a jet area of 4 cm^2^, as described by Gupta et al. (2010)^30^. The droplets were projected at a 15° angle from the mouth at an initial velocity of 21.7 m/s, following findings from Yan et al. (2019)^8^. Detailed human parameters used in the simulations are presented in Table 3.

**Table 3:** Mannequin geometry and parameter settings.

Parameter	Size
Mannequin (L * W * H) (m)	0.5 * 0.2 * 1.73
Distance between left hand and mouth	12 cm
Mouth area	4 cm^2^
Cough jet angle	15°
Jet velocity	21.7 m/s
Cough period	0–0.5 s

This geometric model is crucial for accurately simulating droplet dispersion within a metro cabin. By incorporating realistic dimensions and conditions based on empirical studies, the model ensures that the simulation results are both reliable and relevant to real-world scenarios, providing a faithful representation of droplet dispersion dynamics in a semi-enclosed moving transportation environment.

#### Boundary conditions and meshing

The boundary conditions and meshing protocols employed in this study were meticulously selected to ensure the accuracy and reliability of the results. All cabin walls were subjected to the wall function boundary condition, with the cabin’s ambient air temperature set at 25 °C and relative humidity at 50%. The human body temperature was maintained at 35 °C, and an initial cough jet velocity of 21.7 m/s was used, with the cough injection duration set to 0.5 s as documented by Yan et al. (2019) and Liaqat et al. (2018)^31,32^. For the temporal resolution, the Discrete Phase Model (DPM) was configured with a time interval of 0.2 s, and the total motion duration was modeled over 75 s in 375 time steps.

**Figure 4:**
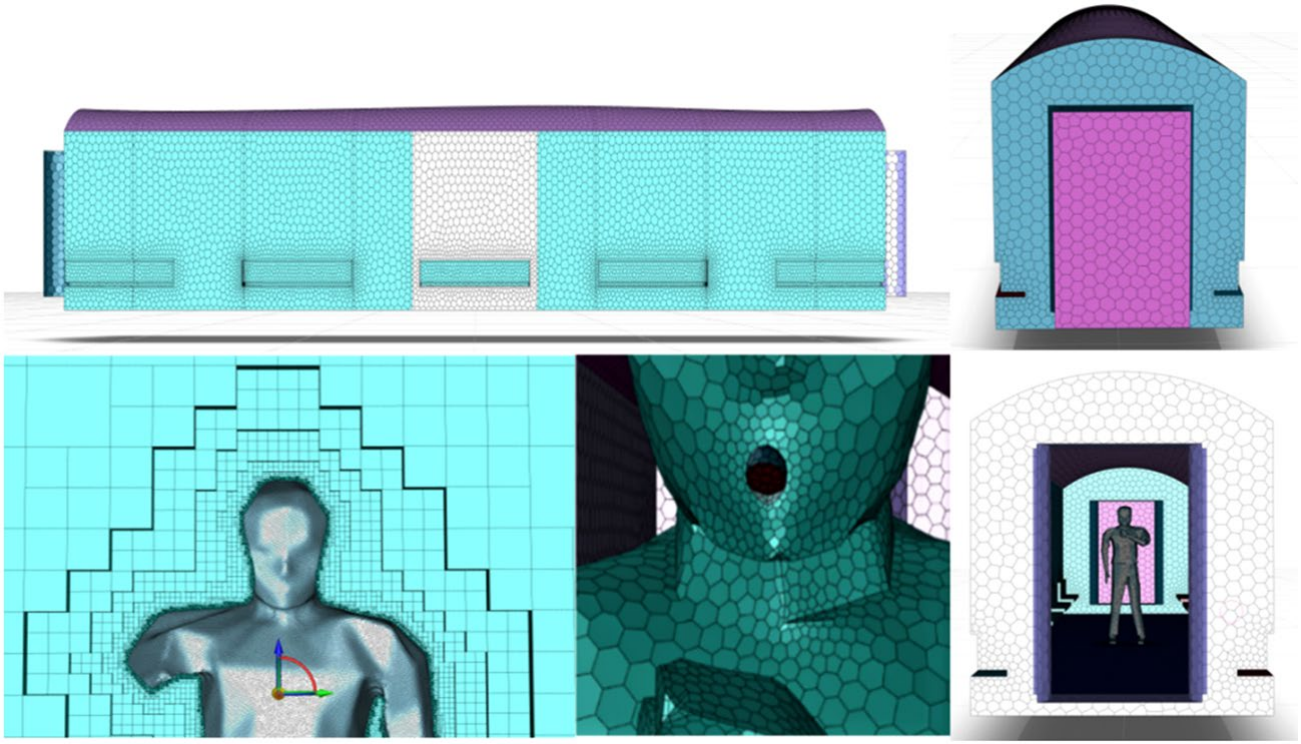
Model meshing.

Finite element meshing, a critical step in numerical simulations, was conducted using ANSYS ICEM CFD 21.0 software^33,34^. The cabin and human body models were discretized into hexahedral cells, with refined poly-hexcore grids near critical areas such as cabin walls, seats, and around the human body and mouth, as depicted in Fig. 4. This approach resulted in a total of 1,157,895 mesh cells for the empty cabin and 2,899,020 for the cabin with the human model.

Within this framework, the cabin was treated as a fixed reference system, presumed to be relatively stationary. The relative velocities between the induced airflow and cabin movement were derived from the data reported by Talaee et al. (2019), highlighting an airflow acceleration phase from 0 to 35s, a constant velocity phase from 35 to 45s, and a deceleration phase from 45 to 75s^28^. The airflow was modeled with a vertical distribution gradient, and disturbances from mechanical ventilation were not considered.

The cough droplets were categorized into six groups based on diameter sizes: 3.5, 6, 20, 50, 112, and 175 μm, which simulate the range of particles produced during respiratory activities like coughing, talking, and breathing. This size range is crucial as it encompasses both fine aerosols and larger droplets, each with distinct behaviors and biological implications. The distribution of coughing droplets, surveyed by Chao et al. (2008), informed these size selections to enhance the model's accuracy in simulating respiratory pathogen transmission and aiding in the development of effective control measures^11^. The simulation findings offer valuable insights into droplet dispersion in a semi-enclosed, moving transportation environment, providing a basis for developing guidelines to ensure commuter safety and health.

### Case studies

**Figure 5:**
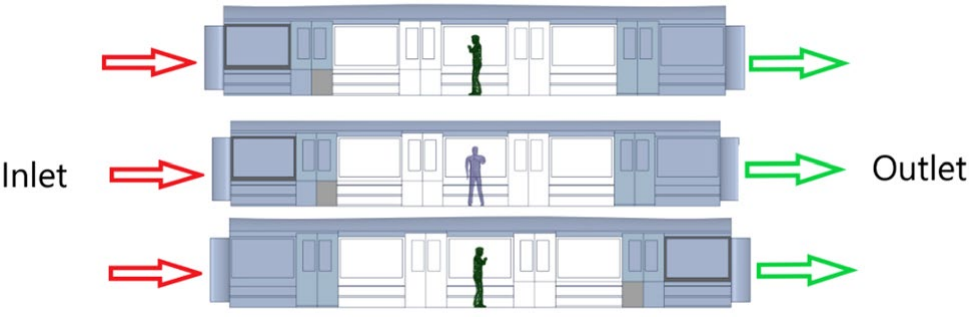
Mannequins with different facing orientations relative to the environment.

To investigate the effects of induced airflow and inertial force on droplet dispersion, three comparative experiment groups were conducted, quantitatively evaluating the role of interior-induced flow, as illustrated in Fig. 5.

1. Experiment group 1 aimed to assess the impact of interior-induced flow on droplet diffusion quantitatively. The group conducted simulated experiments both with and without airflow injection in the cabin during the one-station travel period, which includes acceleration, constant velocity, and deceleration phases. This setup allowed the examination of conditions both with and without the influence of induced airflow and inertial forces.
2. Experiment group 2 aimed to evaluate how the orientation within the cabin affects the diffusion of droplets. The experiments were organized for three different orientations: facing the inlet, the rear, and the side, as depicted in Fig. 5. The aim was to understand the effects of these orientations on droplet dispersion under various working conditions.

**Figure 6:**
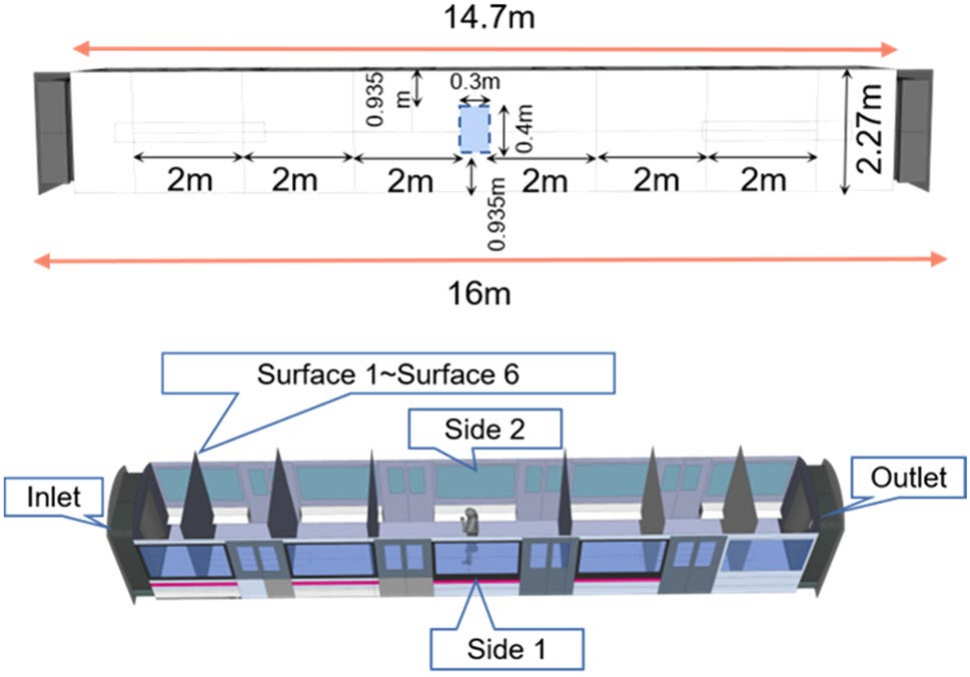
Layout and size of monitoring surface.

Experiment group 3 aimed to better understand the infection risks associated with different cabin locations relative to an infected person. Monitoring surfaces were placed at 2-meter intervals on both sides of the manikin, with three surfaces on each side, making a total of six monitoring surfaces. Each surface measured 2.27 m by 2.62 m (W, H), located as shown in Fig. 6. The study aimed to analyze the infection risk at each surface depending on its orientation to determine relative safety.

These experimental groups provided essential insights into the dispersion of droplets in a semi-enclosed, moving transportation environment. The results highlighted that interior-induced airflow significantly impacts droplet diffusion, and the orientation of the manikin influences infection risk levels. These findings aim to support the development of strategies to reduce infection risks in public transportation, thereby informing guidelines for ensuring commuter safety.

### Infection probability index

To assess infection risk, the study utilized the Watanabe dose–response model, previously applied to various airborne infectious diseases, including severe acute respiratory syndrome-coronavirus disease 2019 (SARS-CoV)^35^. This model estimates the probability of infection, P(n) [%], as a function of the pathogen dose, n^36^. While it is challenging to ascertain the minimum dose of the CoV pathogen required for infection, the study leveraged data from the "Discrete Phase Report" model. This approach enabled the authors to track and count the number of droplets over time at different surface locations, providing a quantitative measure of potential infection risk.

7 $$\begin{array}{c}P\left(n\right)=1-\mathit{exp}\left(n/Ln\left(k\right)\right),n\in \left.0,+\infty \right)\#\end{array}$$

To calculate the infection probability *P*(*n*) [%], we used an exponential expression of the number of discrete droplets n. To normalize this calculation, we took the parameter Ln(*k*) to be − 1e+3. However, the Ln(*k*) parameter is the most challenging parameter, as it depends on various factors such as the number of tracked particles, dilution of ambient exhaled air, deposition losses, pathogen inactivation due to evaporation, convection distance/time from the infectious to the susceptible person, room conditions (relative humidity, temperature, airflow, type of ventilation), anatomical and physiological characteristics of the infectious/susceptible persons, whether or not the infectious person is wearing a face mask (as this significantly affects exhalation flow), and the biological properties of the pathogen. Therefore, it is difficult, if not impossible, to predict the situational risk of infection during a one-to-one exposure in detail. Even if one had an example, the situational variability is substantial, making it hard to generalize superior knowledge. Therefore, detailed examples may not be very helpful in guiding infection control measures.

In summary, the Watanabe dose–response model was used to calculate the infection probability index. However, the Ln(*k*) parameter is challenging to determine accurately due to the complex interplay of various factors. Therefore, it is difficult to predict the situational risk of infection during a one-to-one exposure in detail. Nonetheless, the infection probability index can provide valuable insights into the relative risk of infection at different locations within the cabin.

### Numerical verification

**Figure 7:**
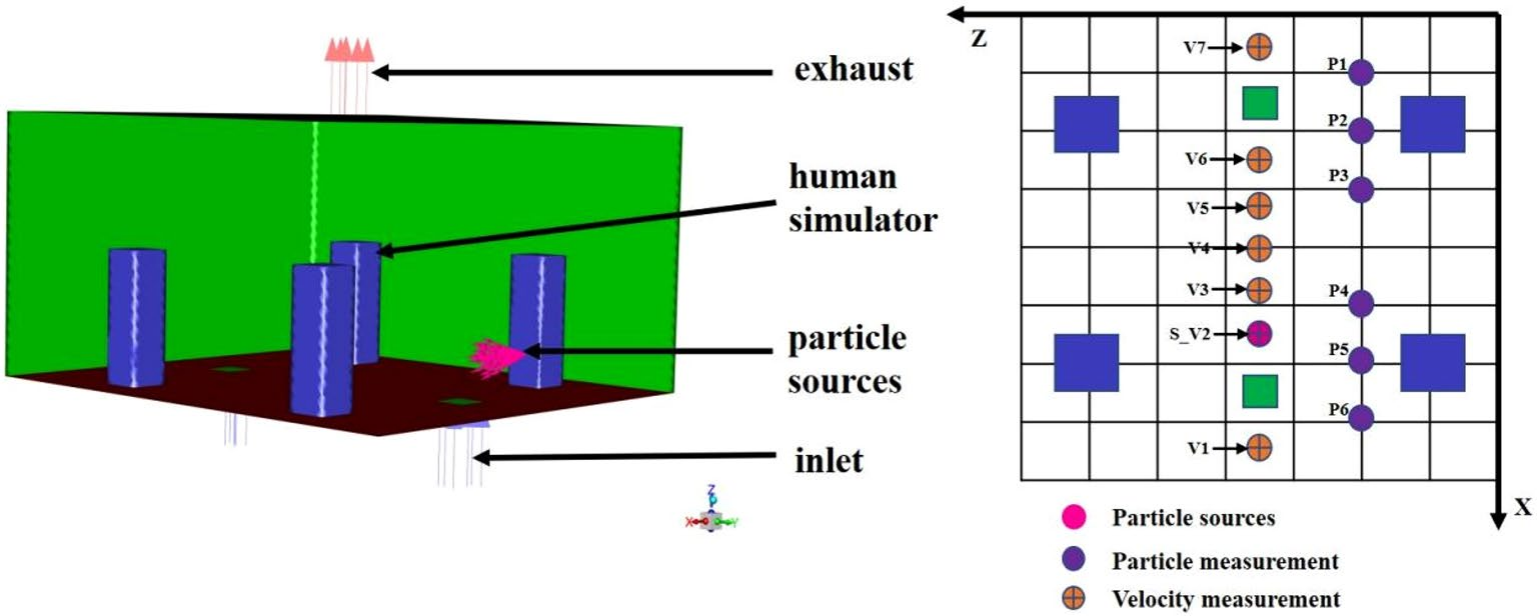
Experimental environment configuration and measuring line layout.

To accurately simulate droplet dispersion under various conditions, the CFD model was validated through a two-phase process, involving both dynamic and static validations. Dynamic validation was conducted using the relative velocity distribution data between the accelerating or decelerating subway train and the internal induced flow, as measured by Talaee et al. (2019)^28^. This phase focused on the relative velocities at different accelerations, which formed the basis for simulating human cough droplet diffusion. Static validation aimed to confirm the accuracy of droplet diffusion modeling; for this, we replicated and compared the experimental data from Li et al. (2022)^37^. The configuration of the indoor environment (shown in Fig. 7) and the physical conditions (detailed in Table 4) were adopted from Zhang and Chen (2006)^38^. The experiments were performed in a full-scale room equipped with an underfloor air distribution system (UFAD). The room featured two air supply outlets located on opposite sides of the ceiling, with inlet directions perpendicular to the floor in the X-direction. The supply airflow was set at 340 m^3^/h. Inside the room, four heated mannequins were positioned, and particles averaging 0.7 μm in diameter were emitted from a source placed 0.3 m above the floor. Air velocity and particle concentration were measured along monitoring lines V1 to V7 and P1 to P6, respectively.

**Table 4:** Experimental environment configuration related parameters.

Parameters	Size
Length	4.8 m
Width	4.2 m
Height	2.44 m
Total supply air flow	340 m^3^/h
Particle source height	0.3 m
Particle source flow rate	6.384 × 10^−2 ^kg/s
Average particle size	0.7 μm

**Figure 8:**
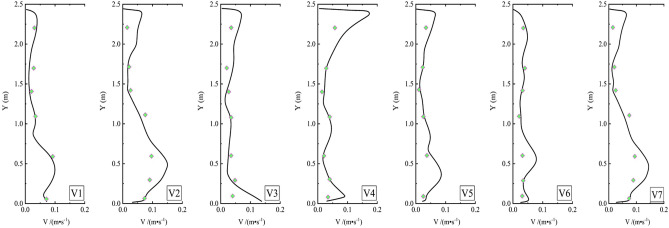
Comparison of simulated and measured velocity in lines V1–V7 (prismatic symbol: measured velocity by Li et al. (2022); solid lines: simulated velocity).

Figure 8 displays the comparison of simulated velocities against experimental data across monitoring lines V1 to V7. The results demonstrated strong consistency between the simulated and experimental data, affirming the accuracy of the numerical simulation method.

**Figure 9:**
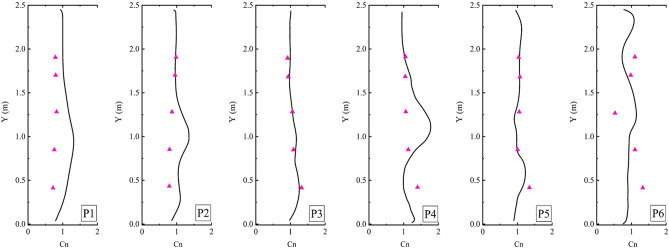
Comparison of simulated and measured particle concentration profiles in lines P1–P6 (triangle symbol: measured concentration by Li et al. (2022); solid lines: simulated.

Figure 9 compares the particle concentrations measured by Li et al. (2022) with experimental data across monitoring lines from P1 to P6. Despite the complex boundary conditions of the internal flow field, the simulation accurately captured the particle concentration distribution at several monitoring lines. Overall, the numerical simulation results aligned well with the experimental data, underscoring the precision and validity of the numerical approach.

In summary, the experimental data from Li et al. (2022)^37^ was instrumental in verifying the reliability of the CFD-based numerical simulation method for modeling indoor pollutant diffusion. The strong agreement between the numerical results and experimental data confirms the accuracy of the simulation approach.

## Results

### Effects of induced wind and inertial force on droplet trajectories

This study quantitatively evaluated the impact of interior-induced airflow on droplet diffusion through comparative simulations with and without airflow injection during a one-station period (acceleration-constant velocity-deceleration phases). As a control variable, the orientation of the human face in these simulations was directed towards the cabin inlet.

During the acceleration phase, particles were carried towards the cabin outlet by the airflow direction, then drifted back towards the cabin inlet during the deceleration phase, affecting the trajectory of smaller-sized particles throughout the cabin.

**Figure 10:**
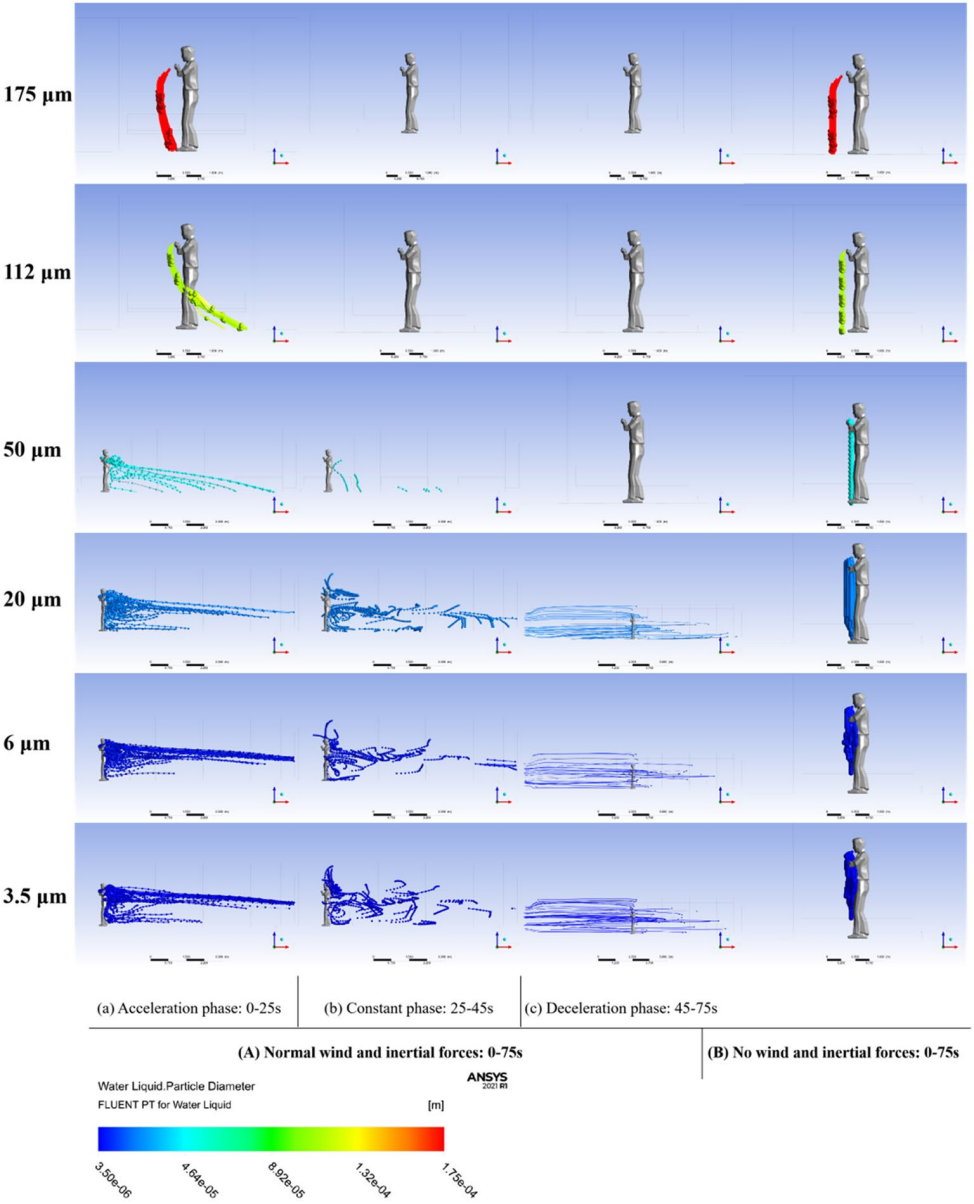
The particle trajectory tracking chart of two scenarios: (**A**) Normal wind and inertial force scenario during 0–75 s; (**B**) No wind and inertial force scenario during 0–75 s. (a), (b) and (c) are the trajectory traces of various particle sizes in the acceleration, constant velocity, and deceleration phases of the cabin, respectively.

Figure 10 illustrates the droplet trajectory-tracking scheme using the Lagrangian method. In scenarios without induced airflow (Fig. 10B), gravity and buoyancy forces predominantly influenced the droplet movement, causing them to move towards the ground post-exhalation in line with resultant forces. Conversely, in the induced airflow scenario (Fig. 10A), droplets were significantly affected by drag force in addition to gravity and buoyancy, resulting in varied trajectories; some droplets followed the airflow while others descended towards the ground.

**Figure 11:**
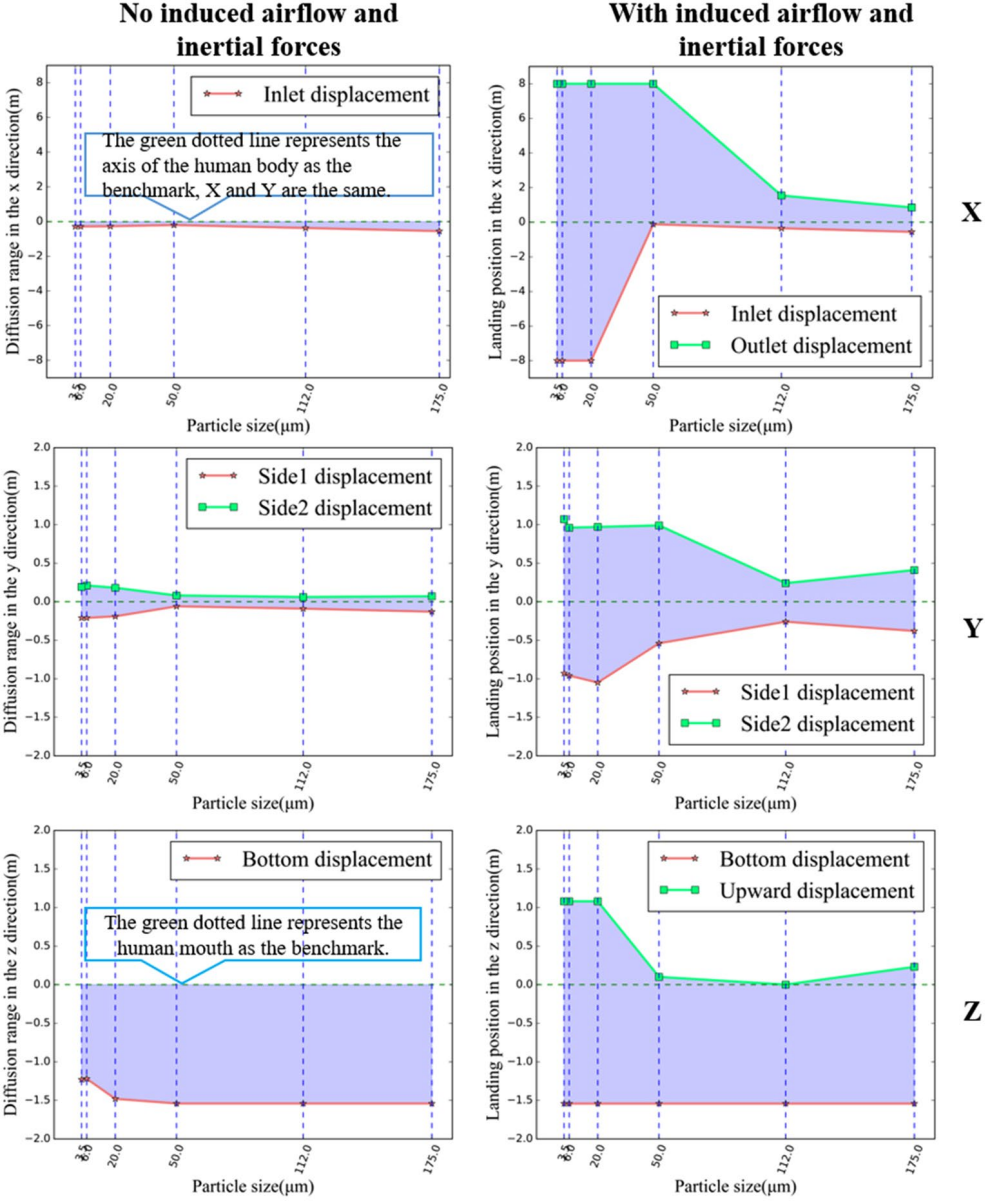
Differences in the diffusion range of each size droplet in the presence or absence of induced airflow and inertial forces, including the range in the X, Y and Z directions respectively. (The X, Y, and Z axes represent the length, width, and height directions of the carriage, respectively, all oriented from the standpoint of a standing human body. Taking the X-axis as an example, the positive direction of the X-axis indicates that droplets from the human body move towards the exit of the carriage, while the negative direction indicates that droplets move towards the entrance of the carriage.)

The simulation results revealed the following: (1) in the no induced airflow case, six sizes of droplets spread within 2 m from the human body in the x-direction, as shown in Fig. 11. The moving direction was the same as the initial injection direction, and some small-sized droplets (3.5, 6 μm) were suspended; (2) in the with induced airflow case, the trajectories of different-sized droplets differed significantly. The large-sized droplets (50, 112, 175 μm) were controlled by gravity and inertial forces and, therefore, fell on the ground quickly, while the small-sized droplets (20, 6, 3.5 μm) were much more guided by the drag force and, therefore, floated throughout the cabin back and forth; (3) during the acceleration phase, the particles spread towards the cabin outlet with the direction of airflow and then drifted towards the cabin inlet during the deceleration phase due to the change of airflow direction, so that the trajectory of small-sized particles was affected by airflow throughout the cabin.

To visually represent these differences, Fig. 11 tabulates the diffusion range of each droplet size in the x, y, and z directions. The displacement range fluctuated significantly with induced airflow, with larger particles showing more directional stability than smaller ones. For instance, 50 μm particles spread up to 7.276 m in the X-positive direction (towards the cabin outlet) under airflow, compared to just -0.27 m without airflow. Smaller particles drifted beyond the cabin outlet, recording displacements as far as 8 m.

**Figure 12:**
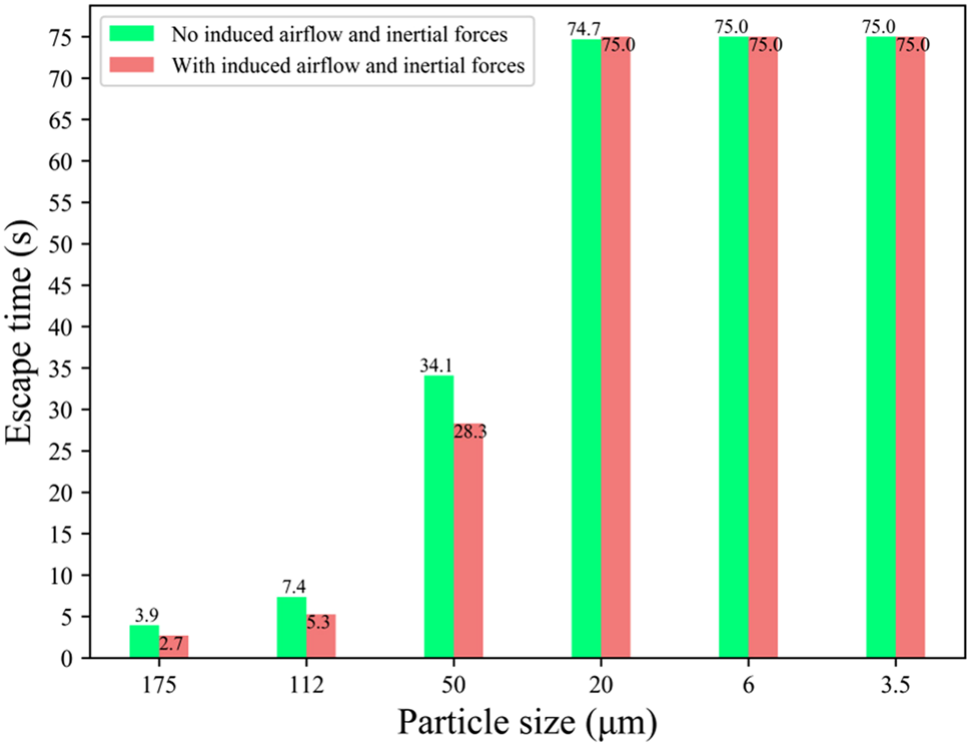
Differences of escape times for droplet sizes with the presence and absence of airflow.

Figure 12 compares the escape times for both scenarios, revealing that larger droplets (175, 112, 50 μm) were less influenced by the airflow compared to smaller and medium-sized droplets (20, 6, 3.5 μm), which remained airborne for up to 75 s without settling.

In summary, the study demonstrates that induced airflow significantly influences the diffusion trajectories of droplets within the cabin, with larger particles exhibiting more stability in displacement and less variation in escape times than smaller particles. These findings provide critical insights into the dynamics of droplet diffusion in indoor environments and are instrumental in developing effective infection control measures.

### Differences in the diffusion trajectories of droplets with three different facing orientations

This experiment explored the impact of the dummy's three facing orientations in the cabin—facing the inlet, the outlet, and side1—on the trajectories of droplets of various sizes, influenced by drag and inertial forces.

**Figure 13:**
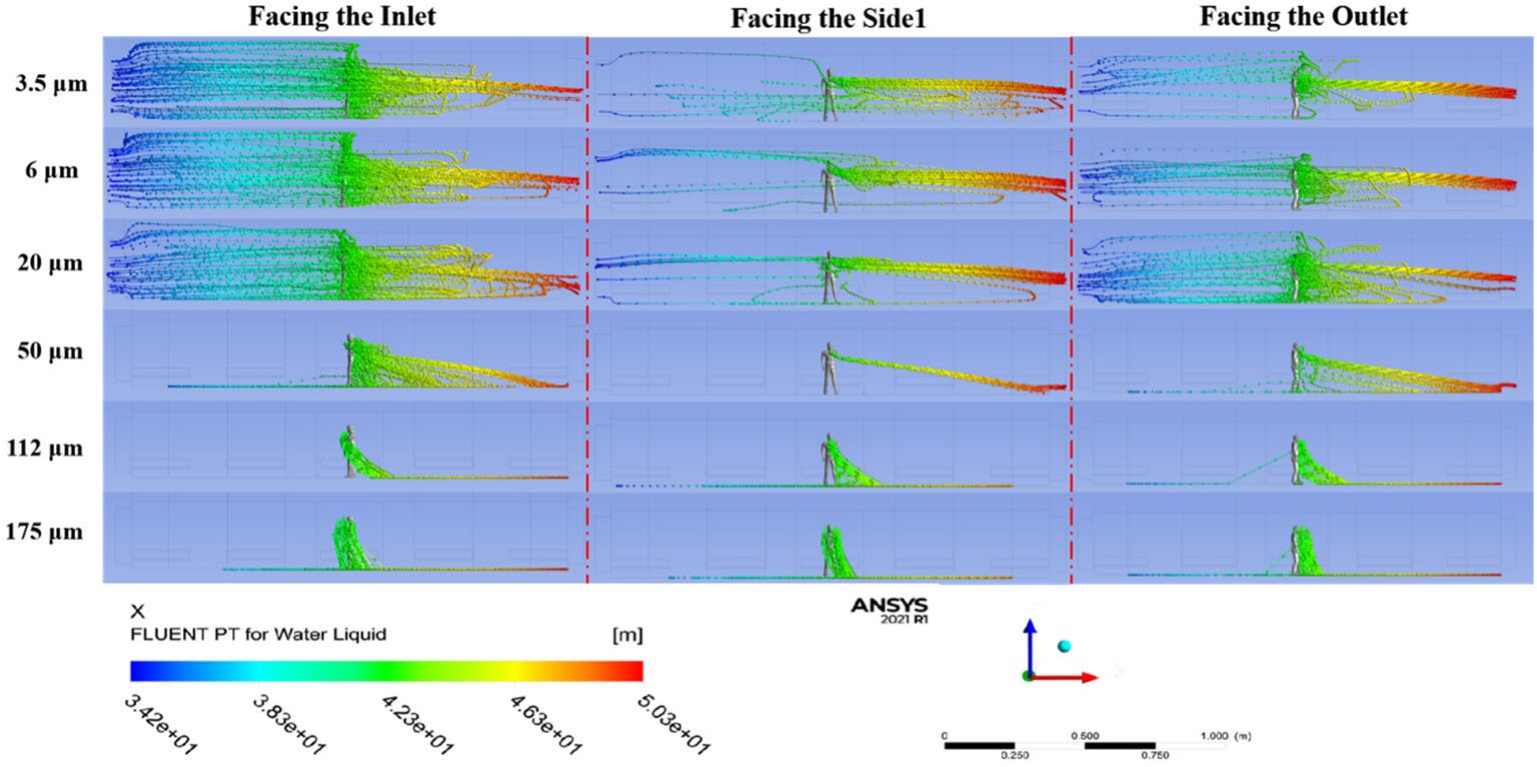
Differences of the diffusion trajectories of droplets with three different facing orientations.

Figure 13 illustrates the droplet trajectory-tracking scheme using the Lagrangian method. Notably, the "facing side1" orientation markedly differs from the other two. In this orientation, as the human body width obstructs the airflow, creating a local vortex, both "facing the inlet" and "facing the outlet" scenarios are less conducive to droplet escape. Conversely, in the "facing side1" scenario, droplets are ejected perpendicular to the airflow direction, allowing medium and small particles to escape further and faster toward the cabin outlet.

**Figure 14:**
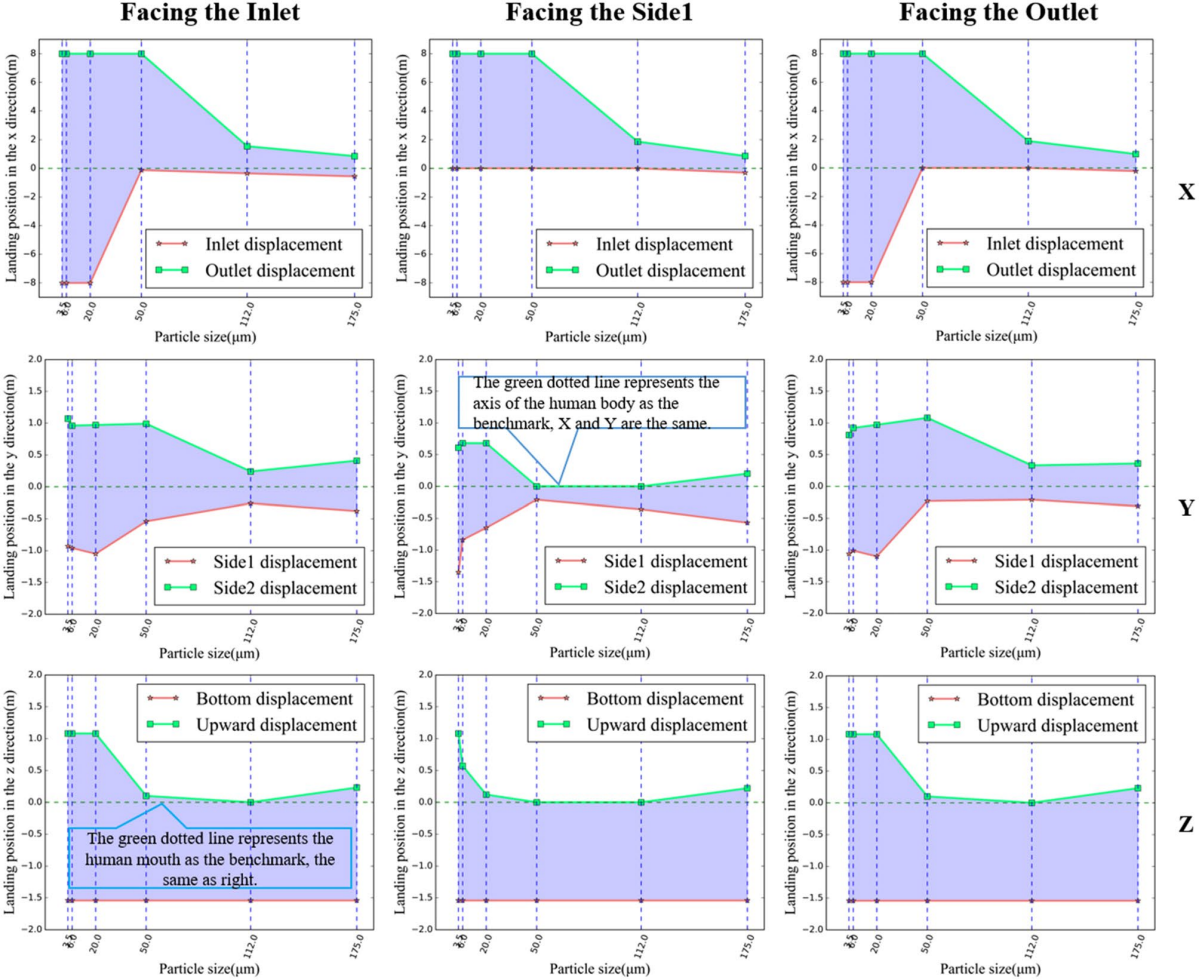
Differences in the diffusion range of each size droplet in three different orientations, including the range in the X, Y and Z directions respectively. (The X, Y, and Z axes represent the length, width, and height directions of the carriage, respectively, all oriented from the standpoint of a standing human body. Taking the X-axis as an example, the positive direction of the X-axis indicates that droplets from the human body move towards the exit of the carriage, while the negative direction indicates that droplets move towards the entrance of the carriage.)

Further insights from Figs. 13 and 14 show that the diffusion direction and distance for large and medium-sized droplets (50, 112, 175 μm) are almost identical across the three orientations. However, different orientations significantly affect the diffusion trajectories for smaller droplets (20, 6, 3.5 μm). During the cabin’s acceleration phase (0–25s), droplets initially follow the airflow towards the outlet and then change direction during the deceleration phase (45–75s), where small-sized droplets achieve a balanced state. Specifically, in the "facing side1" scenario, most droplets escape through the cabin's rear outlet during acceleration, with only a few reversing direction.

**Figure 15:**
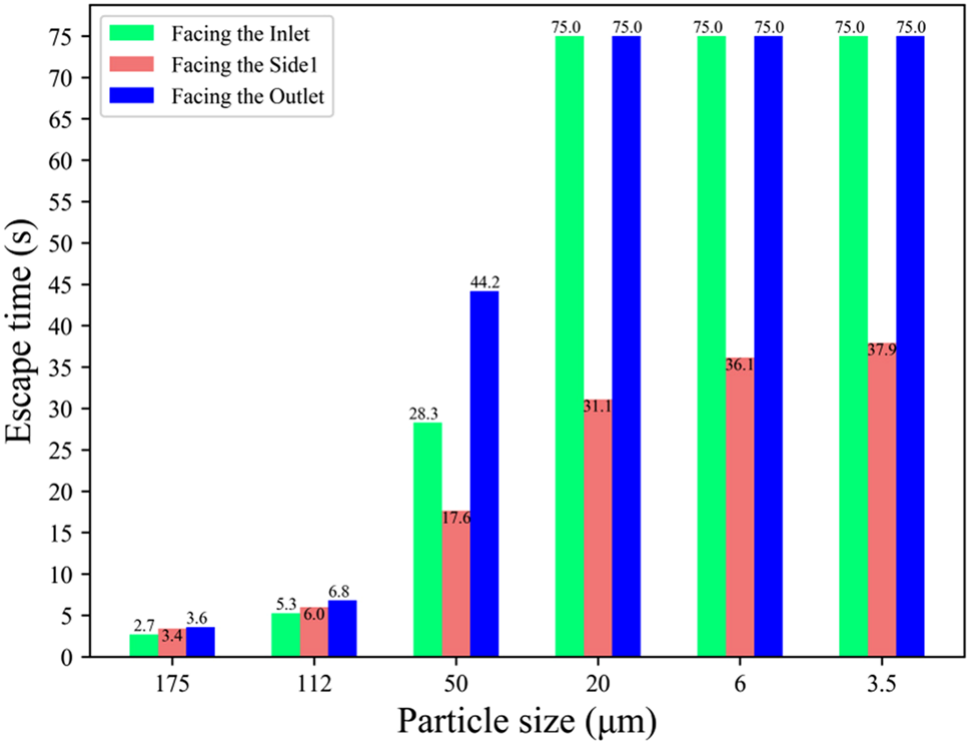
Comparison of escape times in three directions for each particle size.

Figure 15 outlines the escape times for droplets under the three orientations, revealing that escape times are primarily influenced by the orientation of the human body. For small and medium-sized droplets (50, 20, 6, and 3.5 μm), the escape times vary significantly, with obstructions caused by the body in the "facing the inlet" and "facing the outlet" scenarios delaying escape by 10s to 40s. Conversely, large droplets (175, 112 μm), less influenced by airflow, exhibit escape times of around 7s, consistent across all orientations.

Furthermore, there is a fundamental difference between the two cases: the case with airflow is prolonged for the small droplets being carried by the airflow, filling the whole cabin. In contrast, the case without airflow is prolonged, as the small droplets remain suspended in front of the body throughout the period and never land. Therefore, there is a clear difference between the two scenarios regarding the escaping states. The escape time of small particles (3.5, 6 μm) that remained suspended in the air or particle sizes (3.5, 6, 20 μm) spread throughout the cabin without landing on the ground was recorded as 75s.

In summary, the simulation results demonstrated that the orientation of the human body significantly influences both the diffusion trajectory and escape time of droplets within the cabin. The "facing side1" orientation proved most effective for droplet escape, while the "facing the inlet" and "facing the outlet" orientations impeded droplet dispersion. The variability in escape times was pronounced for small and medium-sized droplets, with delays ranging from 10 to 40s, whereas large droplets showed consistent escape times across all orientations. These findings provide valuable insights into the dynamics of droplet diffusion in indoor environments and can inform the development of effective infection control strategies.

### Differences in the infection risks among three orientations

Respiratory diseases are transmissible through tiny airborne droplets. When infected individuals cough or sneeze, they expel droplets containing saliva, mucus, or other bodily fluids that can transmit infections upon contact. This study aimed to identify high-risk areas for transmission within a confined space, using a color-coded system to denote varying levels of risk. The injector, measuring 0.2 × 0.4, was positioned at distances of 2 m, 4 m, and 6 m from six monitoring surfaces aligned from left to right and labeled S1 to S6.

**Figure 16:**
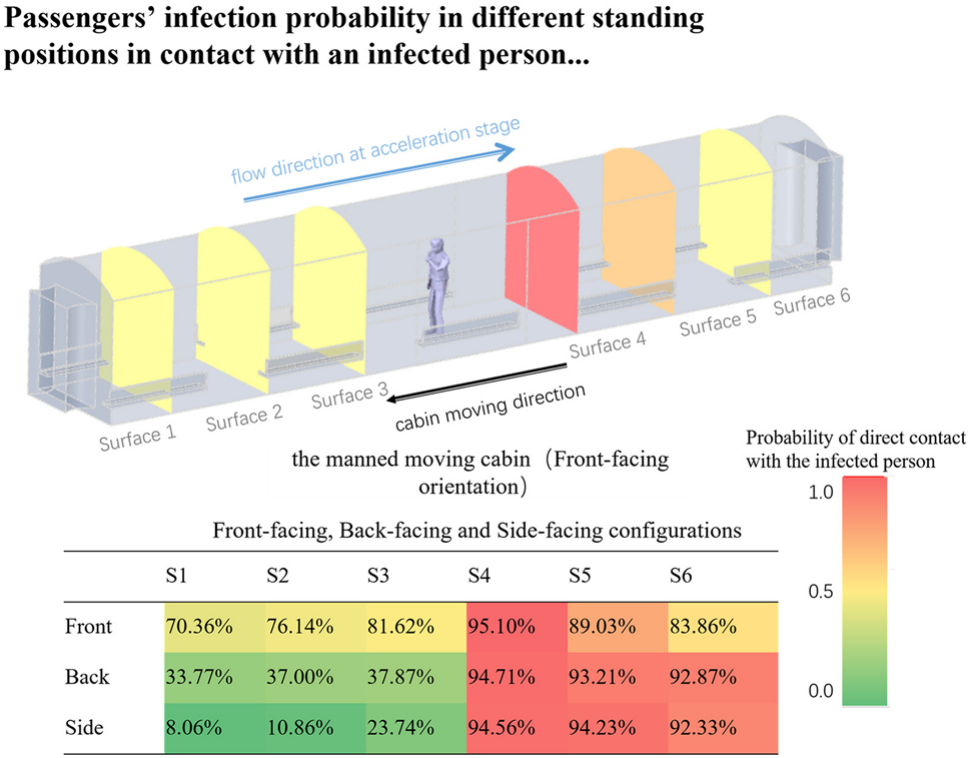
Front-facing, back-facing and side-facing configurations.

Based on the findings in Fig. 16, the outlet is the most likely position to become infected, regardless of the orientation of the face. Only three monitoring positions in the side-facing configuration are considered safe, using a criterion of a safety probability of below 30%. This conclusion is surprising since it goes against the conventional belief that areas behind an infected person's back are safe. The reasons are complex. Firstly, it is mainly because the induced flow in the cabin can change the direction of droplet motion, even for large-sized droplets. Secondly, the cabin-induced flow in the acceleration and deceleration stages are opposite, which leads to an increase in the risk to the whole space in the cabin. A part of the droplets that do not flow out of the cabin in the first stage becomes a later pollution source in the third stage. Thirdly, considering the wind shadow area size, the human body becomes an obstacle to the spread of droplets, slowing down the movement of droplets and providing pollution for the reverse movement of droplets in the later stage. This result can be observed in both the outlet-facing and side-facing configurations.

In conclusion, the study reveals that the outlet is the likeliest position for infection transmission, regardless of face orientation. The traditional assumption that the area behind an infected person remains safe is debunked by the complex dynamics of droplet movement within indoor spaces. The induced airflow in the cabin significantly influences droplet trajectories, with the cabin’s airflow patterns during acceleration and deceleration phases amplifying the risk throughout the cabin. Furthermore, the human body can obstruct and slow the movement of droplets, contributing to pollution in subsequent movement phases. These insights are critical for developing effective infection control measures in indoor environments.

## Discussion

This study was designed to investigate the impact of induced airflow combined with inertial forces on the dispersion of cough droplets. The insights gained are crucial for identifying safe positions—in terms of both direction and distance—relative to a potentially infected individual. These findings will aid in guiding epidemic prevention strategies within semi-enclosed environments.

### The effect of induced airflow on the diffusion of cough droplets

Research by Luo et al. (2022) demonstrated that the risk of infection is closely tied to ventilation rates; with low ventilation, droplets tend to disperse only locally within the cabin, whereas higher ventilation airflows cause droplets to spread more extensively^6^. Similarly, Fan et al. (2022) how different-sized particles behaved at wind speeds of 1.54 m/s (low speed) and 6.68 m/s (high speed), finding that the risk of infection was minimal at lower speeds but significantly increased at higher speeds due to strong airflows carrying small and medium-sized droplets into breathing zones^39^.

In this study's Group 1 experiments, in a still-air environment, droplets of all sizes were observed to hang suspended and settle within a 2-m radius around the human body throughout the entire motion cycle. In contrast, in environments with time-varying winds, changes in wind direction caused smaller droplets (3.5, 6, 20 μm) to move throughout the carriage. Assuming a train with 8–12 semi-enclosed carriages, the circulating airflow would carry these droplets toward the carriage exit during acceleration, and then back again with the remaining evaporated pathogen nuclei during deceleration.

In conclusion, this study provides critical insights into how induced airflow affects the dispersion of cough droplets in indoor environments, particularly within semi-enclosed transit settings. The findings from Luo et al. (2022) and Fan et al. (2022) underscore the significant role of ventilation rate and wind speed in managing the spread of respiratory diseases. Together, these studies highlight the intricate dynamics of droplet diffusion and emphasize the necessity for comprehensive infection control strategies in indoor settings^6,39^.

### Effect of different orientations on droplet dispersion

In this study, Groups 2 and 3 demonstrated that the orientation of a person's face within the cabin significantly influences the trajectories and dispersion ranges of droplets of various sizes. Specifically, the side-facing orientation resulted in unidirectional droplet dispersion, markedly increasing infection rates on one side of the cabin. In the "side1" scenario, droplets exited the cabin in the shortest time, primarily affecting individuals directly downstream. This observation underscores the role of induced airflow in accelerating droplet spread to adjacent cabins, thereby heightening infection risks if an infected individual faces sideways.

Previous research supports that droplet spread is strongly influenced by the circulation and magnitude of wind^40–42^. Given the dense networks of metro rails, particularly highlighted during the COVID-19 pandemic, the potential for widespread contamination is considerable.

Consequently, it is recommended that the public wear KN95 masks and present a negative nucleic acid test taken within the last 48 h when using public transportation. Although maximizing ventilation is a common strategy, it can paradoxically increase infection risks if not managed properly. Ventilation practices should ideally be conducted before or after passenger loading periods, and any individuals showing symptoms of infection should be promptly directed to designated isolation areas.

## Limitations

This study makes a significant contribution to the field of infectious disease cybernetics and offers scientific support for epidemic transmission models within complex networks. However, to enhance the understanding of droplet dispersion in metro environments, future research should address certain limitations noted in this work.

First, the current study models viral nuclei as particles encapsulated within droplets, without accounting for the processes of fragmentation and evaporation, or the effects of local airflow disturbances such as lift and vortices caused by body heat plumes and human respiration. Future research should comprehensively investigate these factors, examining how droplet fragmentation, evaporation, and thermal plumes influence droplet behavior.

Second, this research does not account for the impact of widely used "maximum mechanical ventilation" settings and varied ventilation strategies (e.g., up in and down out, down in and up out, up in and up out) on droplet dispersion. Incorporating these elements into future studies would provide a more detailed understanding of how ventilation influences airborne pathogen spread.

Third, the study utilized a single human body model to simulate droplet dispersion. However, real-world scenarios often involve varying human postures and densities, from standing and leaning to sitting, both densely and sparsely arranged. Future studies should examine droplet dispersion across different passenger densities, categorized as sparse, moderate, and dense, to mirror actual conditions more closely.

In conclusion, while this research provides essential insights into the effects of induced airflow on cough droplet dispersion in semi-enclosed transit settings, it also highlights the need for further investigation. Addressing the limitations outlined above will enable future studies to offer a more comprehensive understanding of droplet dynamics in indoor environments.

## Conclusions

The objective of this study was to investigate the effect of induced airflow doubled with the inertial force on cough droplet dispersion. Through CFD simulations, we analyzed the aerodynamic diffusion behavior of cough droplets from one passenger in subway cabins during acceleration-constant velocity-deceleration phases and detected safe standing positions. The following conclusions were drawn from our study.

We found that the escape times of large and small droplets were significantly different in Figs. 12 and 15. When the induced flow absence, large droplets were the first to hit the ground, resulting in a short escape time, while small droplets remained near the body throughout the cycle. Due to their lighter weight, small droplets lose moisture in the air and become aerosols. In the induced airflow environment scenarios, small droplets also had an escape time of more than 75s as they spread in the direction of the airflow. Morawska (2006) has previously shown that droplet size significantly affects its ability to disperse, deposit, and carry pathogens^43^. Figure 11 and Fig. 14 indicate that large and small droplet sizes have opposite transport and deposition patterns. Large droplets are quickly deposited to the ground by gravity, while small droplets lose moisture in the air and become viral nuclei, carrying the pathogen to more distant locations. Figure 14 also shows that viruses spread over 7m due to airflow effects. Therefore, maintaining a social distance is the most effective non-medical policy against viral invasion, but for outdoor or indoor airflow cases, it is not sufficient. In this study, we found that the traditional 1–2 m social distance is inadequate.

Furthermore, we simulated three orientations of human standing and calculated infection rates at six monitoring surfaces to identify relatively safe locations. Figure 16 shows that the infection rates for the six monitoring surfaces were normally distributed for both the inlet and outlet-facing scenarios, with the highest infection risk at S4, the closest point to the body, and decreasing at the sides. The side-facing case was unique, as the majority of droplets escaped to the next carriage in the initial direction of the airflow, which took only 38s for 8 m, as the airflow was perpendicular to the initial direction of the droplets, and the width of the body did not block the droplets from spreading.

In summary, our study showed that induced airflow in carriages exacerbates the spread of the virus, rendering the safe distance guideline no longer applicable. For metro cabins and other dynamic transport environments, it is crucial to test for the presence of infected persons before entering the train. It is recommended that everyone wear KN95 masks when traveling, wash hands frequently, sanitize regularly, and take full protective measures. It is also advisable to ventilate the carriages carefully, especially to avoid the significant spread of the virus due to the negative effect of mechanical ventilation in metro cabins. The results provided insights into the potential transmission of respiratory diseases in semi-enclosed moving transportation environments and could be used to develop guidelines for creating a safe and healthy environment for commuters.

## Data Availability

The datasets and materials used and/or analyzed during the current study are available from the corresponding author on reasonable request.
